# A population-based cohort study on sun habits and endometrial cancer

**DOI:** 10.1038/sj.bjc.6605149

**Published:** 2009-06-23

**Authors:** E Epstein, P G Lindqvist, B Geppert, H Olsson

**Affiliations:** 1 Department of Obstetrics and Gynaecology, Institute of Clinical Sciences Lund, Lund University Hospital, 221 85 Lund, Sweden; 2 Clintec, Department of Obstetrics and Gynaecology, Karolinska University Hospital, Huddinge, 14186 Stockholm, Sweden; 3 Department of Oncology, Institute of Clinical Sciences Lund, Lund University Hospital, 221 85 Lund, Sweden

**Keywords:** endometrial cancer, lifestyle, risk factor, sun habits, sun bed use, vitamin D

## Abstract

**Background:**

No large cohort study has examined the risk of endometrial cancer in relation to sun exposure.

**Methods:**

A population-based cohort study of 29 508 women who answered a questionnaire in 1990–92, of whom 24 098 responded to a follow-up enquiry in 2000–02. They were followed for an average of 15.5 years.

**Results:**

Among the 17 822 postmenopausal women included, 166 cases of endometrial cancer were diagnosed. We used a multivariate Cox regression analysis adjusting for age and other selected demographic variables to determine the risk of endometrial cancer. Women using sun beds >3 times per year reduced their hazard risk (HR) by 40% (0.6, 95% confidence interval (CI) 0.4–0.9) or by 50% when adjusting for body mass index or physical activity (HR 0.5, 95% CI 0.3–0.9), and those women who were sunbathing during summer reduced their risk by 20% (HR 0.8 95% CI 0.5–1.5) compared with women who did not expose themselves to the sun or to artificial sun (i.e., sun beds).

**Conclusion:**

Exposure to artificial sun by the use of sun beds >3 times per year was associated with a 40% reduction in the risk of endometrial cancer, probably by improving the vitamin D levels during winter.

Excess weight alone is estimated to cause around 50% of all endometrial cancer cases in Europe and the United States (Calle and Kaaks, 2004). Among the cohort studies of lifestyle factors and endometrial cancer, no large study has investigated the effect of UV exposure on risk. Sunlight is known to be the most important source of vitamin D. A Spanish study found an inverse correlation between endometrial cancer mortality and sun exposure (indirectly assessed by using latitude as an index of solar UVB levels) (Grant, 2007), whereas a recent meta-analysis adding the results of three case–control studies showed no correlation with dietary intake of vitamin D of which sunlight is the most important source (McCullough *et al*, 2008).

This prospective cohort study was carried out to assess the potential influence of sun and sun bed exposure on the risk of endometrial cancer.

## Materials and methods

The ethics committee of Lund University (LU-632-03) approved the study. A total of 1000 women from each age group between 25 and 64 years, in all 40 000 women, without prior diagnosis of cancer, were chosen from the general population registry of the South Swedish Health Care Region by computerised random selection. The women were invited to fill out a written questionnaire aiming to identify risk factors for endometrial cancer. Of the women invited, 29 508 (74%) agreed to participate; those who had had a hysterectomy before recruitment were excluded. The initial survey was performed in 1990–92 and a follow-up, similar to the first, in 2000–02. Of the women answering the first questionnaire, 24 098 (82%) also completed the second questionnaire. The initial survey covered reproductive history, hormone use, family history of gynaecological cancer, constitutional factors, marital status, level of education, and lifestyle factors such as level of physical exercise, smoking habits, and sun exposure habits. The follow-up survey covered the past medical history, body mass index (BMI), and present regular exercise.

Low-potency oestrogen use and hormone treatment use were classified as previously described (Epstein *et al*, 2009). For simplicity, we choose not to present the effect of hormone-use duration in this paper. In our previous study, we found that the use of only combined hormone therapy was related to a decreased risk of endometrial cancer (odds ratio (OR) 0.3, 95% confidence interval (CI) 0.1–0.8), whereas the protective effect was found after 2 years, and increased with extended duration of use. In women using only high- or low-potency oestrogens, the overall risk was doubled (OR 2.3, 95% CI 1.5–3.7), being highest during the first 2 years, and dropping slightly thereafter (Epstein *et al*, 2009). Use of combined oral contraceptives (COCs) was dichotomised into those who had ever used and those who had never used COCs at the inception of the study. Women not answering the question were assumed to be never users. As information on menopause age was missing in quite some women, we decided not to analyse this demographic variable.

Parity was divided into null parity or parity (i.e., one or several children) as we found no difference in one, two, or multiparous. Smoking habits were recorded at the inception of the study, categorised as never smokers (reference) or ever smokers. The wording of the sun exposure questions at study inception was: (i) do you sunbathe during summer? (never, 1–14 times, 15–30 times, >30 times); (ii) do you go abroad on vacation to swim and sunbathe? (never, once every year or two, once a year, two or more times a year); (iii) do you use a sun bed? (never, 1–3 times, >4 times per year). The first two questions were dichotomised into yes or no in the final analysis.

The BMI (weight (kg) per length (m^2^)) was calculated on the basis of the present length and weight reported in the second questionnaire. Body mass index was classified into three groups (BMI <25, normal weight; BMI 25–29, over weight; or BMI >29, obese). The level of present physical exercise was also obtained at the second questionnaire, and it was divided into three categories: none, go for a walk one or more times a week, or strenuous exercise weekly.

By using the unique identification number assigned to all Swedish residents, all deaths and causes of death were established through the National Cause of Death Registry (NCD). Endometrial cancers were identified through the Swedish Cancer Registry (South Swedish Regional Tumour Registry and National Swedish Tumour Registry), using the ICD9 code numbers 179, 182, and the ICD 10 code number C54. Women with sarcoma of the uterus were excluded. Vital status and the diagnosis of endometrial cancer were determined up until 31 December 2007.

## Statistics

Only postmenopausal women were included in this analysis, performed with Cox regression using 95% CIs (Cox, 1972). Endometrial cancer was used as a dependent variable and ‘time at risk’ as the time variable. The time at risk was calculated from the day of recruitment up until the date of cancer diagnosis, death, or last day of follow-up (31 December 2007), whichever came first. Age adjustment was performed in all analyses, as increasing age is a strong risk factor for endometrial cancer. We also made multivariate models adjusting for age, civil status, educational level, COCs use, hormone use, family history of gynaecological cancer, physical activity, and BMI. All calculations were performed using SPSS software (Statistical Package for Social Sciences (SPSS, Inc., Chicago IL, USA), version 10.0.1) and *P-*values <0.05 were considered significant.

## Results

At the end of the follow-up in December 2007, 166 of the 17 822 postmenopausal women had developed endometrial cancer, and demographic characteristics in relation to their risks are presented in Table 1. Decreased risk was found in women ever using COCs. Ever use of any combined hormone replacement therapy decreased the risk, whereas ever use of low- or high-potency oestrogen only doubled the risk. Ever smokers had a decreased risk of endometrial cancer (hazard risk (HR) 0.7, 95% CI 0.5–0.99). An increased risk was found in women overweight (BMI 25–<30) or obese (BMI⩾30) women, and among those with a family history of gynaecological cancer (Table 1).

Table 2 shows the multivariate analysis of sun bed use, sun exposure, and of physical exercise in relation to risk with adjustment for age, civil status and educational level, hormone use, and family history of gynaecological cancer. Adjustment for other selected variables from the inception of the study did not change the HR substantially. Women using a sun bed had a significantly decreased risk (HR 0.7, 95% CI 0.5–0.9), the reduced risk being confined to those using sun beds >3 times per year (HR 0.6, 95% CI 0.4–0.9). Sunbathing during the summer reduced the risk by 20%, whereas traveling abroad to sunbathe did not affect risk. As compared with women living a sedentary life, women doing strenuous exercise every week had a decreased risk of endometrial cancer (HR 0.5, 95% CI 0.3–0.98). In Table 3, we present the effect of sun bed use in a multivariate Cox regression analysis adjusting for age, civil status, and education, in combination with variables obtained at the second questionnaire: present physical activity (Model 1) and BMI (Model 2). The point estimates and CIs for sun bed users were approximately the same when adjusting for physical activity or BMI. The overall wider CIs in Table 3 reflect the large number of missing cases; we lack information on present physical activity in 6139 women and BMI in 4490 women.

## Discussion

A novel finding is that women using sun beds had a decreased risk of endometrial cancer, a dose-dependent effect with a 40% reduced risk among those using a sun bed >3 times per year.

There is indirect evidence that UV exposure might affect the risk of endometrial cancer, women living at higher latitudes having a lower risk than those at lower latitudes (Grant, 2007; Mohr *et al*, 2007). In our study, we found that sunbathing abroad or in Sweden during the summer had little or no effect on the risk, whereas sun bed users had a significantly reduced risk. The major source of vitamin D is sunlight, which penetrates the skin and converts 7-dehydrocholesterol to 25-hydroxicholecalciferol vitamin D_3_ (25-OHVitD) through pre-vitamin D (Holick *et al*, 2008). Sweden is a Nordic country with a scarcity of sunlight during the winter and therefore many women will develop seasonal vitamin D-deficiency, with low levels during winter. Sun beds are commonly used during winter in Sweden. Sun bed users (UVB light) have been shown to have good vitamin D levels (Holick, 2007). Sun beds with UVA light also produce a small amount of UVB light sufficient to increase vitamin D levels (Porojnicu *et al*, 2008; Thieden *et al*, 2008). Vitamin D deficiency has also been associated with a 30–50% increased risk of colon, prostate, and breast cancer, along with an increased mortality in these cancers (Holick, 2007). It has been suggested that if a cell become malignant, 1,25-dihydroxy Vitamin D can induce apoptosis and prevent angiogenesis, thereby reducing the potential for the malignant cell to survive (Holick, 2006). However, a recent meta-analysis of three case–control studies showed no relation between dietary vitamin D intake and endometrial cancer (McCullough *et al*, 2008). This can probably be explained by sunlight being the most important source of vitamin D, most diets being low in Vitamin D. In this meta-analysis, the highest intake category was 244 to 476 IU per day (McCullough *et al*, 2008), which is much less than the recommended 2000 IU per day (Garland *et al*, 2007). An ecological study produced an estimated overall reduction in cancer mortality of 20% for Western European women living below the 59th latitude and of 9% for women in the United States if they were all given a daily dose of 1000 IU vitamin D (Grant *et al*, 2007). The disadvantage of using sun beds to reduce the risk of endometrial cancer is the reportedly increased risk of melanoma (OR 1.8, 95% CI 1.2–2.7) in regular sun bed users (Westerdahl *et al*, 1994). Another alternative to reduce the risk of endometrial cancer and other vitamin D-related malignancies would be to provide adequate (2000 IU per day) vitamin D supplement in countries such as Sweden where people are at risk of developing seasonal vitamin D deficiency. We would welcome new prospective randomised studies on the health benefits of vitamin D supplement in populations at risk of vitamin D deficiency.

The positive effect of artificial sun exposure through sun bed use also continued when adjusting for potential confounding lifestyle factors such as BMI and physical activity.

Overall, physical activity reduced the risk of endometrial cancer by half. Other cohort studies have shown differing results; most have found a decreased risk in physically active women (Furberg and Thune, 2003; Schouten *et al*, 2004; Friberg *et al*, 2006), whereas others showed no association (Colbert *et al*, 2003). Hypothesised mechanisms include changes in obesity and/or fat mass as well as a reduced exposure to endogenous oestrogens (McTiernan *et al*, 1998). As information on present physical activity was obtained at the second survey and was lacking in a substantial number, the related findings must be interpreted with caution.

In our study, smoking women had a decreased risk of endometrial cancer, which is in agreement with other prospective cohort studies (Terry *et al*, 2002; Viswanathan *et al*, 2005; Al-Zoughool *et al*, 2007; Loerbroks *et al*, 2007).

We found that sun bed users were at lower risk of endometrial cancer and there may be an association between vitamin D deficiency and endometrial cancer.

## Figures and Tables

**Table 1:** Demographic characteristics of postmenopausal women with and without endometrial cancer

	**Women with cancer (*n*=17 656)**	**Women without cancer (*n*=166)**	**Hazard ratio**	**95% CI**
*Data from inception of the study*				
Education				
<9 years	4232	47	1.1	0.7–1.6
9 years	2393	22	0.9	0.5–1.5
10–12 years	3126	26	1.0	0.6–1.6
>12 years	5384	49	1.0	Reference
Other	2521	22	0.9	0.5–1.5
Marital status				
Unmarried	898	4	0.5	0.2–1.4
Married	13 762	126	1.0	Reference
Divorced	1914	16	0.9	0.5–1.5
Widow	1082	20	1.8	1.1–2.9
Smoker				
Never smoker	7491	87	1.0	Reference
Ever smoker	10 041	77	0.7	0.5–1.0
Parity				
0	1990	15	1.3	0.8–2.2
⩾1	15 666	151	1.0	Reference
Used combined oral contraceptives				
Never	8525	105	1.0	Reference
Ever	9131	61	0.5	0.4–0.7
Use of hormone theraphy^a^				
Never use	11 431	100	1.0	Reference
Ever use combined HT	3388	19	0.7	0.4–1.1
Ever use oestrogen only	2484	42	1.9	1.3–2.7
Family history of gynaecological cancer				
No	17 067	153	1.0	Reference
Yes	589	13	2.5	1.4–4.4
				
*Data from the second survey*				
BMI				
<25	7278	45	1.0	Reference
25–29	4392	41	1.4	0.9–2.2
>29	1540	36	3.5	2.2–5.4
Missing information	4446	44	1.7	1.1–2.5

BMI=body mass index; CI=confidence interval; TH=hormone therapy.

a Use of hormones was gathered at both questionnaires and the combined result is presented.

Cox regression analysis with age adjustment was used throughout.

**Table 2:** Sun habits, sun bed use, and physical exercise with regard to risk of endometrial cancer: multivariate Cox regression analysis

	**Women with cancer**	**Women without cancer**	**HR**	**95% CI**
*Data from inclusion 1990–1992*				
Use of sun beds				
No	10 319	112	1.0	Reference
Yes	6947	46	0.7	0.5–0.98
1–3 times per year	2324	18	0.9	0.5–1.5
>3 times per year	4623	28	0.6	0.4–0.9
Sun bathing during summer				
No	1240	16	1.0	Reference
Yes	15 985	147	0.8	0.5–1.5
Sunbathing abroad during vacation				
No	7671	72	1.0	Reference
Yes	9792	93	1.0	0.7–1.4
				
*Data from follow-up interview 2000–02*				
No	1007	14	1.0	Reference
A walk 1–2 times a week	6472	59	0.7	0.4–1.3
Strenuous exercise	4058	28	0.5	0.3–1.1

CI=confidence interval; HR=hazard ratio.

COX regression analysis used with adjustment for age, education, marital status, hormone use, and family history of gynaecological cancer.

**Table 3:** Sun bed use and risk of endometrial cancer, multivariate analysis with adjustment for age, BMI, or physical exercise

			**Model 1**		**Model 2**	
	**Women with cancer**	**Women without cancer**	**HR**	**95% CI**	**HR**	**95% CI**
*Use of sun beds*						
No	10 319	112	1.0	Reference	1.0	Reference
Yes	6947	46	0.6	0.4–0.96	0.6	0.5–0.9
1–3 times per year	2324	18	0.8	0.4–1.4	0.8	0.4–1.4
>3 times per year	4623	28	0.5	0.3–0.9	0.5	0.3–0.9

BMI=body mass index; CI=confidence interval; HR=hazard ratio.

Model 1 COX regression analysis adjusted for age, education, marital status, and physical activity.

Model 2 COX regression analysis adjusted for age, education, marital status, and BMI.
